# Stabilizing and Anti-Repressor Elements Effectively Increases Transgene Expression in Transfected CHO Cells

**DOI:** 10.3389/fbioe.2022.840600

**Published:** 2022-05-26

**Authors:** Qin Li, Rui-Fang Yan, Yong-Xiao Yang, Chun-liu Mi, Yan-long Jia, Tian-Yun Wang

**Affiliations:** ^1^ School of Basic Medicine, Xinxiang Medical University, Xinxiang, China; ^2^ International Joint Research Laboratory for Recombinant Pharmaceutical Protein Expression System of Henan, Xinxiang Medical University, Xinxiang, China

**Keywords:** Chinese hamster ovary cell, stabilizing and anti-repressor elements, transgene expression, epigenetic regulatory elements, green fluorescent protein

## Abstract

Chinese hamster ovary (CHO) cells are currently the most widely used host cells for recombinant therapeutic protein (RTP) production. Currently, the RTP yields need to increase further to meet the market needs and reduce costs. In this study, three stabilizing and anti-repressor (SAR) elements from the human genome were selected, including human SAR7, SAR40, and SAR44 elements. SAR elements were cloned upstream of the promoter in the eukaryotic vector, followed by transfection into CHO cells, and were screened under G418 pressure. Flow cytometry was used to detect enhanced green fluorescent protein (eGFP) expression levels. The gene copy numbers and mRNA expression levels were determined through quantitative real-time PCR. Furthermore, the effect of the stronger SAR elements on adalimumab was investigated. The results showed that transgene expression levels in the SAR-containing vectors were higher than that of the control vector, and SAR7 and SAR40 significantly increased and maintained the long-term expression of the transgene in CHO cells. In addition, the transgene expression level increase was related with gene copy numbers and mRNA expression levels. Collectively, SAR elements can enhance the transgene expression and maintain the long-term expression of a transgene in transfected CHO cells, which may be used to increase recombinant protein production in CHO cells.

## Introduction

Recombinant therapeutic proteins (RTPs, antibodies) are an important part of the biotechnological drugs produced (Overton, 2014; Rodgers and Chou, 2016). The mammalian cell line is the most used biopharmaceutical production platform, which is due to the ability to produce proteins with post-translation modifications that are similar to human natural proteins (Zhu, 2012; Dalton and Barton, 2014; Portolano et al., 2014). Chinese hamster ovary (CHO) cells are the most used mammalian expression system, which has become the most important expression system for the production of RTPs and vaccines (Betts and Dickson, 2017; Kuo et al., 2018; Valente et al., 2018). The establishment of transfected cell lines with high-level expression of the gene of interest (GOI) is often an arduous process due to the heterogeneity of transgene expression. To obtain stable and highly transfected GOI cell lines, cell line engineering, growth medium and production process modification, and expression vector design were performed (Jia et al., 2018; Guo et al., 2020; Wang et al., 2022). Among these strategies, the expression vector design is an effective method to increase GOI expression level and stability.

In previous studies, some epigenetic regulatory elements were found to improve the GOI yield and stability of clones in CHO cells. The most common epigenetic elements are the locus control regions, matrix attachment regions (MARs), insulators, ubiquitously acting chromatin opening elements (UCOEs), and stabilizing and anti-repressor elements (SARs) (Buceta et al., 2011; Pfaff et al., 2013; Harraghy et al., 2015; Guo et al., 2020). These elements can be cloned into different sites of the expression vector, such as the flank of the expression box, upstream of the promoter, or downstream of poly A, which can improve the transcription efficiency and stability, to significantly reduce the time and cost required to produce many recombinant proteins. The MAR, UCOE, promoter, intron, insulator, selective marker, and poly A can be used to increase GOI expression levels in CHO cells (Mariati Yeo et al., 2014; Chen et al., 2017; Zhao et al., 2017; Tian et al., 2018; Xu et al., 2018; Li et al., 2019; Wang et al., 2022). MAR can increase the expression level in CHO cells and reduce the variation of transgene expression among transformants (Wang et al., 2008; Wang et al., 2010; Wang et al., 2012; Li et al., 2013; Zhao et al., 2017; Li et al., 2018; Tian et al., 2018; Li et al., 2019).

The ability of epigenetic elements to protect transgenes from epigenetic changes caused by changes in the cell culture environment is an important feature of these elements because it contributes to the long-term, stability, and high-level expression of proteins. Inserting epigenetic regulatory elements into expression vectors can improve the production level of recombinant proteins and the stability of mammalian cell lines.

SARs are regulatory elements that can block the repressor proteins and are highly conserved between the human and mouse, either at the functional or sequence level. Kwaks et al. (2003) screened SAR elements from the human genome library and flanked them in transgene genes and transfected them into mammal cells. They found that SARs can achieve more efficient GOI production (Kwaks et al., 2003). Although several SARs have been shown to increase transgene expression and expression stability in CHO cells (Otte et al., 2007; van Blokland et al., 2007), there are no systematic studies about the effect of SARs on transgene expression level and stability and the mechanism of function is unclear. In this study, three SAR elements were cloned upstream of the promoter of the expression vector, and their effect and mechanism on the transgene on CHO cells were investigated.

## Materials and Methods

### Vector Construction

**FIGURE 1:**
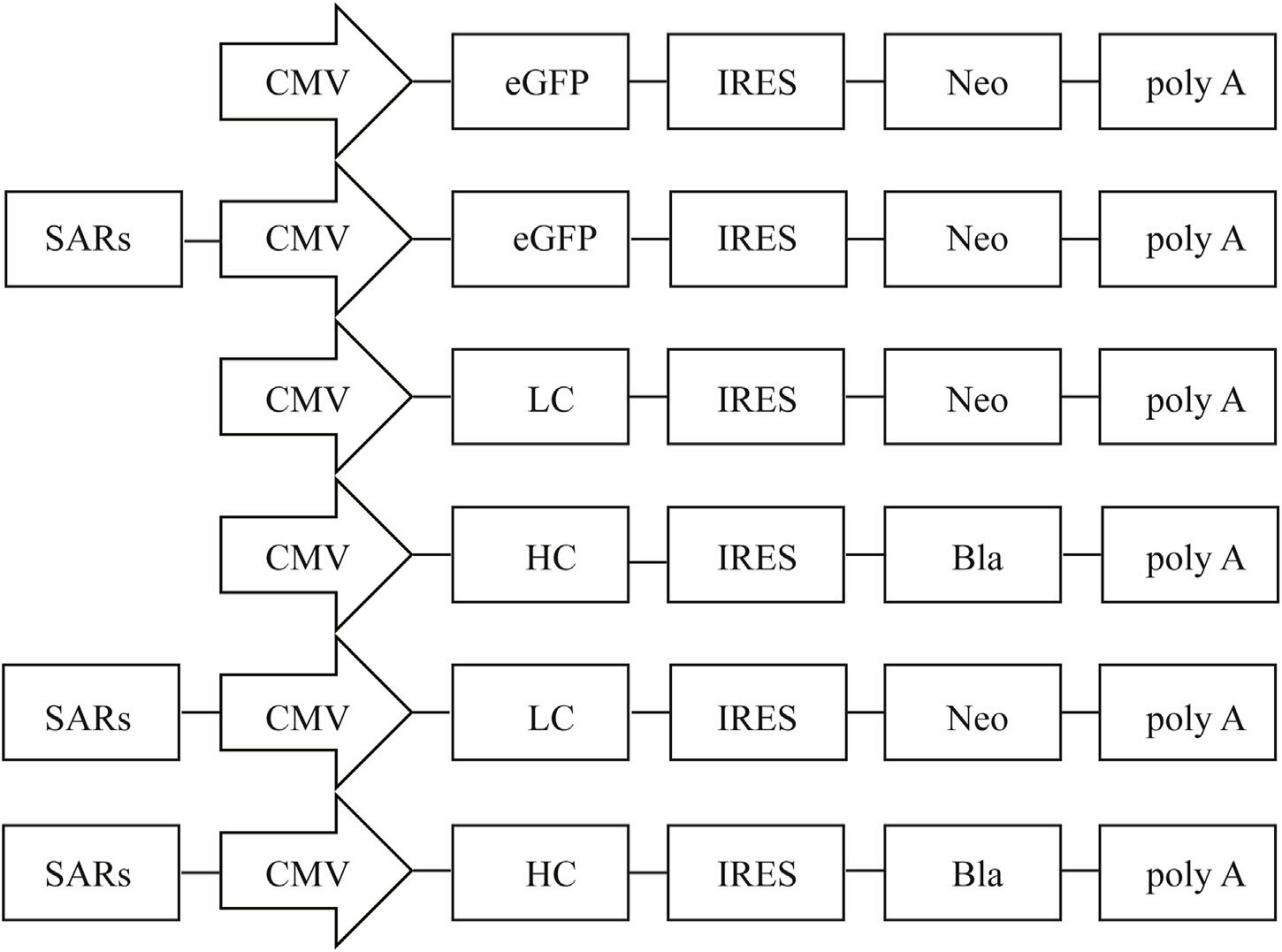
Representation of the vectors containing different SARs. Schematic illustration of the expression vector containing different SARs which were inserted upstream of CMV. CMV, human cytomegalovirus IE gene promoter; eGFP, enhanced green fluorescence protein; IRES, internal ribosome entry site; poly A, polyadenylation signal. Neo, neomycin; Bla, blasticidin.

pIRES-neo vector (Clontech, Mountain View, CA, United States), a bicistronic vector driven by the CMV promoter, was used for the backbone vector. The enhanced green fluorescent protein (eGFP) from pEGFP-C1 (Clontech) was subcloned into pIRES-neo to generate the pIRES-EGFP vector. The human stabilizing and anti-repressor element 7 (SAR7) (GenBank no: AY190751.1, position 1-2101), stabilizing and anti-repressor element 40 (SAR40) (GenBank no: AY190756.1, position 1-1031), and stabilizing and anti-repressor element 44 (SAR44) (GenBank no: AP000525.1, position 8205-9790) were artificially synthesized by General Biosystems (Chuzhou, China). All SARs elements were digested by NruI/BstBI enzymes, and sub-cloned into the upstream of the CMV promoter in the pIRES-EGFP vector according to the molecular standard operation, thereby producing pIRES-SAR7, pIRES-SAR40, and pIRES-SAR44 (Figure 1). The resultant vectors were verified through enzyme digestion and sequencing. To study the effect of SAR elements on the production of recombinant proteins, an expression vector containing the adalimumab heavy chain (HC) and light chain (LC) was constructed. To obtain the stably transfected mAb cell colonies, the LC and HC were coded on two separate plasmids, both carrying the different antibiotic resistance gene (Figure 1). Briefly, the eGFP gene was replaced with the LC and HC of adalimumab gene upstream of the promoters in pIRES-EGFP, pIRES-SAR7, and pIRES-SAR40 to construct the expression vectors pIRES-LC, pIRES-HC, pIRES-LC-SAR7, pIRES-HC-SAR7, pIRES-LC-SAR40, and pIRES-HC-SAR40.

### Cell Culture and Transfection

CHO cells (#A11557-01; Life Technologies, Carlsbad, CA, United States) were cultured in Dulbecco’s Modified Eagle’s Medium/Nutrient mixture F12 (Gibco, Carlsbad, CA, United States) with 10% fetal bovine serum (FBS; Gibco) at 37°C with 5% CO_2_. The cells were counted and inoculated in 24-well plates at a density of 4 × 10^5^ cells/well. After reaching 80–90% confluence, the cells were transfected with pIRES-SAR7, pIRES-SAR40, or pIRES-SAR44; pIRES-EGFP was used as a control vector. Transfections were performed using the Lipofectamine^®^ 2000 reagent (Invitrogen, Waltham, MA, United States) according to the manufacturer’s manual. All transfections were performed in triplicates.

### Transient Expression

At 48-h post transfection, the transfection efficiency of each expression vector was observed under a fluorescence microscope (ECLIPSE Ti; Nikon, Tokyo, Japan), and mean fluorescence intensity (MFI) was detected by flow cytometry as previously described (Wang et al., 2019; Wang et al., 2022). Briefly, a total of 100,000 fluorescent events were obtained using a 530/15 bandpass filter for the green fluorescent signal, which was acquired with an emission wave length of 530 nm. Data acquisition and analyses were performed using Flow Jo software 7.6 (Tree Star, Ashland, OR).

### Screening of Stably Transfected Cells

At 48-h after transfection, cells were cultured in the presence of 800 μg/ml G418 (Invitrogen) for approximately 14 days until the non-transfected control cells were eliminated. Next, transfected cells were seeded (4 × 10^5^ cells/mL) in 6-well plates with 400 μg/ml G418 to produce the stably screened cells for determination of the fluorescence intensity. The eGFP expression was evaluated through flow cytometry using 1 × 10^6^ CHO cells, and non-transfected cells were used as the negative control. Three stably transfected pools were generated for each vector. To analyze the expression long-term stability of the transgene, the stable cell lines were cultured in the absence of antibiotics selection pressure for 120 generations. At generations 30, 60, 90, and 120 after transfection, cells were collected and the eGFP expression level was analyzed through flow cytometry.

### Quantitative Real-Time Polymerase Chain Reaction Analysis

Genomic DNA was extracted from 5 × 10^6^ cells according to the manufacturer’s manual (Beyotime, Shanghai, China). The DNA concentration and purity were determined. Total RNA was extracted using the TRIzol reagent (Sigma, San Francisco, CA, United States) following the manufacturer’s protocols. The isolated RNA was treated with RNase-free DNaseI (Thermo Scientific, Waltham, MA, United States) to eliminate DNA contamination. cDNA synthesis was performed according to the manufacturer’s protocol (Cwbio, Jiangsu, China). Quantitative real-time PCR (qRT-PCR) was performed to determine the eGFP mRNA levels, and gene copy numbers using the SYBR Premix Ex Taq (Takara Bio, Beijing, China). The reaction mixture (10 μL) consisted of 5 μL SYBR Green (TAKARA, Dalian, China), 4 μL template DNA (0.05 μg/μL), 0.3 μL each of the forward and reverse primers (10 nmol/ml), and 0.4 μL deionized water. The glyceraldehyde phosphate dehydrogenase (GAPDH) gene was used as an endogenous control. The eGFP and GAPDH primers were designed (Table 1) and qRT-PCR was performed using an ABI 7500 Fast real-time PCR instrument (Applied Biosystems, Foster City, CA, United States) and the results were analyzed by 7500 Fast System SDS Software. The cycling parameters were as follows: 95°C for 3 min, followed by 35 cycles of 94°C for 30 s, 50°C for 30 s, and 72°C for 30 s. The relative eGFP expression and eGFP copy number were determined with the 2^-∆∆Ct^ method (Livak and Schmittgen, 2001). The experiment was repeated three times.

**TABLE 1:** Sequences of the primers for quantitative real-time PCR.

Gene	Direction		Sequences (5´→3′)
eGFP	Forward		CTA​CGT​CCA​GGA​GCG​CAC​CAT​CT
eGFP	Reverse		GTT​CTT​CTG​CTT​GTC​GGC​CAT​GAT​AT
GAPDH	Forward		CGA​CCC​CTT​CAT​TGA​CCT​C
GAPDH	Reverse		CTC​CAC​GAC​ATA​CTC​AGC​ACC

### Expression of Adalimumab

The adalimumab LC and HC constructs were co-transfected at a 2:1 ratio, and a 4 µL Lipofectamine 2000 transfection reagent per 2 µg plasmid DNA was transfected into each well. After co-transfection, cells were selected in the presence of G418 as described above and Blasticidin (15 μg/ml) to establish stable CHO cell colonies. Then, the transfected CHO cells were suspension-cultured in a serum-free medium (ProteinEasy Biological Co., Ltd., Xinxiang, China) using a working volume of 30 ml in 125-ml Corning shake flasks at 37°C with 5% CO_2_. After 7 days, supernatants were collected and the expression level of adalimumab was analyzed through Western blot.

The cell supernatant transfected with the expression vector was collected and adalimumab expression was determined through western blot. The cell supernatant containing adalimumab and 5× buffer were mixed and boiled at 100°C for 1 min. First, the resolved proteins were transferred onto a PVDF membrane at 25 V for 90 min. The membrane was blocked with non-fat dry milk (NFDM) in TBS with 0.05% Tween-80 (TBS-T) for 1 h, and incubated with the secondary antibody at 1:8000 dilution (dilution buffer, 1% NFDM in TBS-T) (Catalogue no: E031610-01, Goat Anti-Mouse IgG, EarthOx, San Francisco, CA, United States) for 1 h, and washed three times with TBST. The membrane was soaked in ECL chemiluminescent solution and developed under a gel imaging system. At the same time, adalimumab expression was quantified with ELISA according to the provided protocol (ELK Biotechnology Co., Ltd., Wuhan, China).

### Statistical Analysis

Data were analyzed with the SPSS 18.0 software (SPSS Inc., Chicago, IL, United States) using independent sample *t*-tests and reported as the mean ± standard deviation. *p* < 0.05 was considered statistically significant.

## Results

### Transfection Efficiency and Transient Expression

**FIGURE 2:**
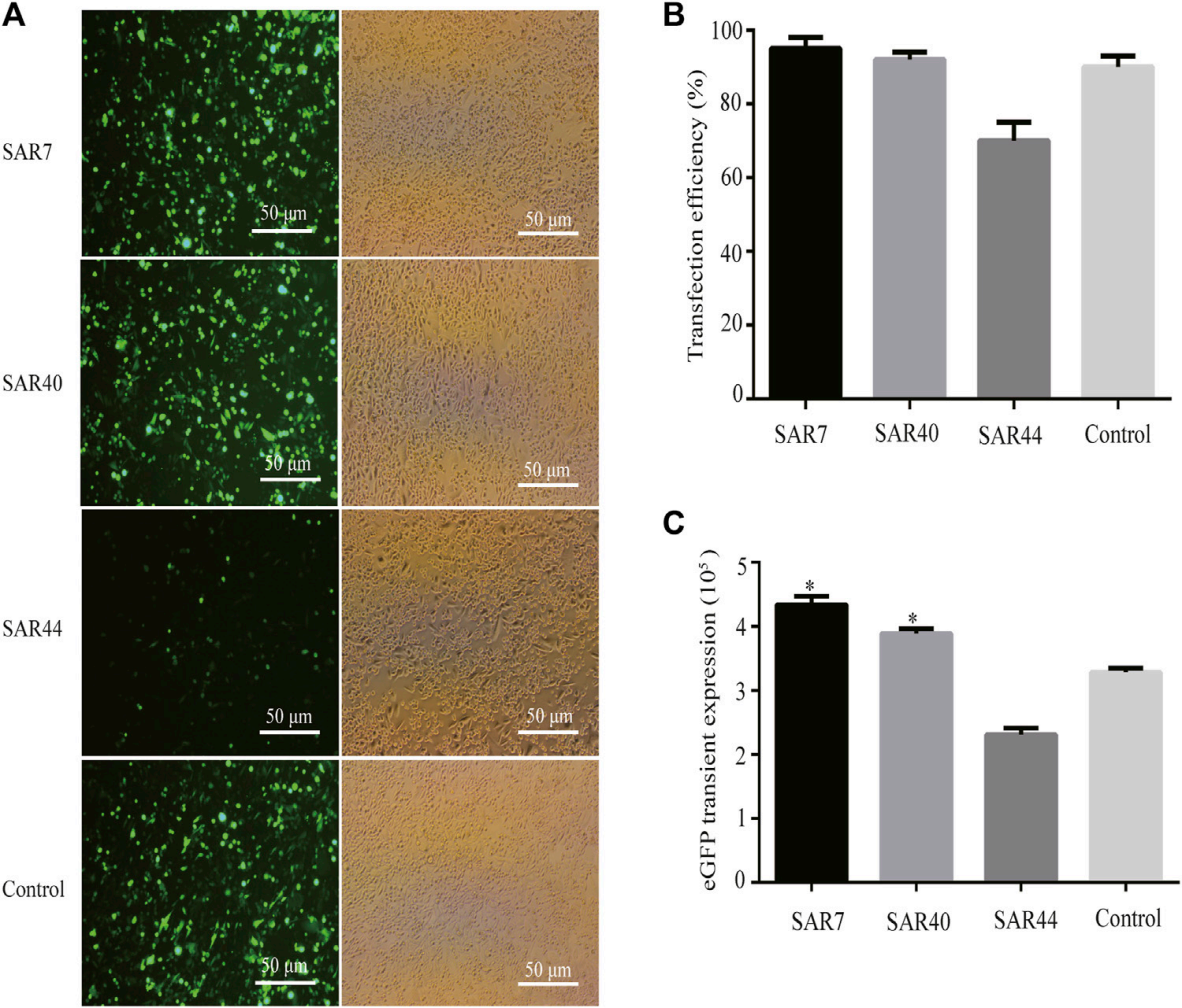
Transient expression of recombinant protein in transfected pools of different SARs. Three vectors were transfected into CHO cells, 48 h after transfection, and the transfection efficiencies were estimated. **(A)** The representative photos were observed by fluorescence microscopy. **(B)** Analysis of the transfection efficiency. **(C)** Analysis of the eGFP transient expression. The results are the mean values obtained for three independent experiments, and the standard deviation is indicated (*p < 0.05).

We first evaluated the effect of different SARs on the transfection efficiency using the eGFP reporter gene. The transfection efficiency and transient eGFP expression levels in CHO cells were observed through a fluorescence microscope 48 h after transfection. Our results showed that the eGFP expression in the vectors containing SAR7 and SAR40 were significantly higher than that of the control vector, while the eGFP expression mediated by SAR44 was weaker than that of the control vector (Figure 2A). The transfection efficiencies of SAR7, SAR40, and SAR44 were approximately 95, 92, and 70%, respectively (*p* < 0.05; Figure 2B), which were consistent with the transgene expression level. Compared with the control, SAR7, SAR40 increased the eGFP transient expression 1.32 and 1.19-fold, but SAR44 did not (Figure 2C).

### Transgene Expression Levels in Stably Transfected Cells

**FIGURE 3:**
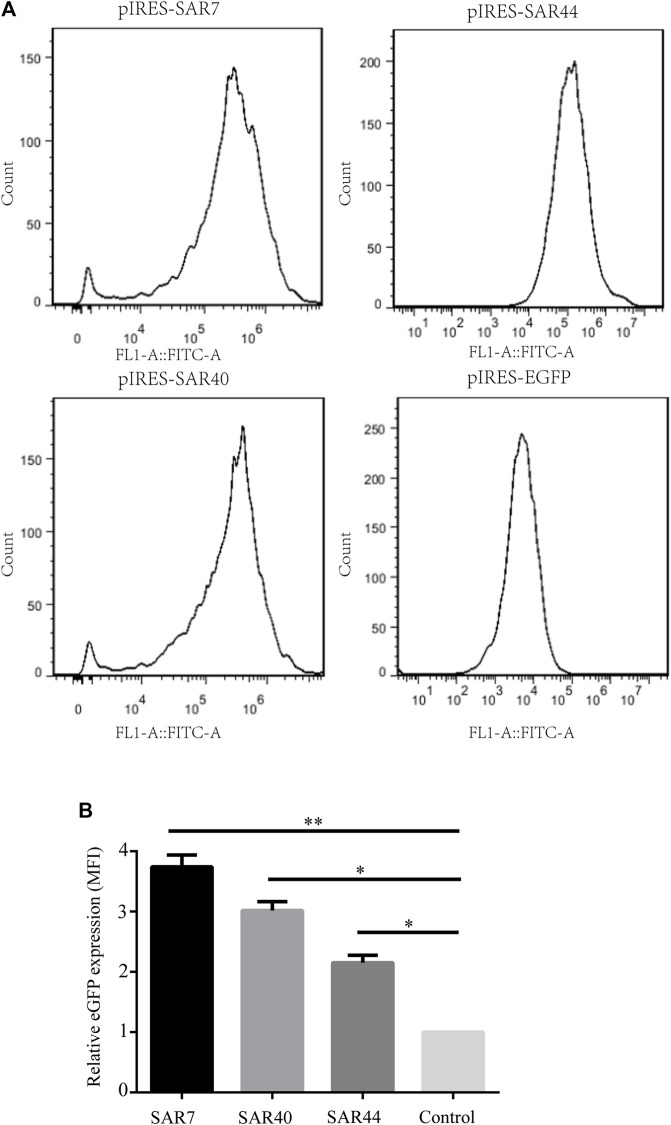
Analysis of MFI in stably transfected CHO cell line. **(A)** The MFI of eGFP of the different construct was measured by flow cytometry on day 20 under G418 selection. **(B)** Fold statistical analysis results of eGFP expression levels in the different constructs. The results are the mean values obtained for three independent experiments, and the standard deviation is indicated (*p < 0.05; **p < 0.05).

To establish stable transfectants, CHO cells were transfected with the respective vectors and subjected to G418 selection; the eGFP expression was further measured through flow cytometry (Figure 3A). Compared with the control vector, the mean fluorescence intensity of eGFP containing SAR7, SAR40, and SAR44 was 3.74-, 3.02- and 2.15-fold higher, respectively (Figure 3B), suggesting SARs can improve transgene expression levels in stably transfected cells.

**FIGURE 4:**
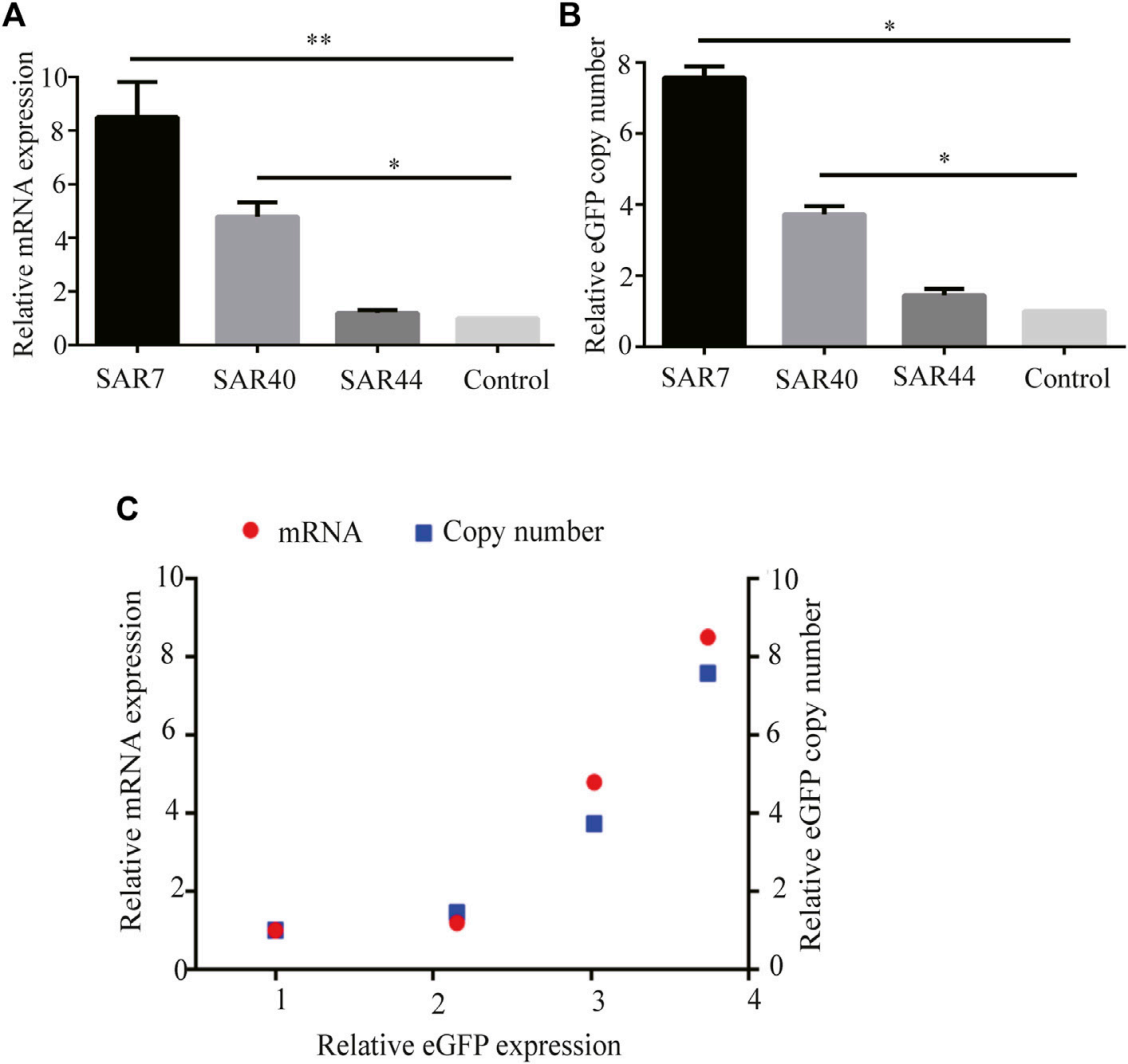
Analysis of the relative eGFP mRNA levels and eGFP copy number in stably transfected CHO cells. After screening under G418, the stable transfected CHO cell pools were acquired. **(A)** Total RNA was isolated and cDNA synthesis was performed, then the mRNA levels were detected using qRT-PCR. **(B)** Relative eGFP gene copy numbers were detected by qRT-PCR. **(C)** Correlation analysis between the relative eGFP copy (blue square), mRNA expression (red) and relative eGFP expression in long-term stable transfected CHO cells. Results were obtained from three independent experiments. The results are the mean values obtained for three independent experiments, and standard deviation is indicated (*p < 0.05; **p < 0.05).

Subsequently, we performed qRT-PCR with cDNAs prepared from cells transfected with SARs and found that SARs increased the eGFP mRNA level compared with the control vector. SAR7, SAR40, and SAR44 increased the eGFP mRNA expression by approximately 8.5-, 4.8-, and 1.2-fold, respectively (Figure 4A).

### Gene Copy Number Analysis

To investigate the relationship between the transgene expression level and gene copy number, we performed qRT-PCR using genomic DNA. The results showed that gene copy numbers in cells transfected with SAR7, SAR40, and SAR44 expression vectors were 7.58-, 3.73- and 1.45-fold higher than that of the control vector (Figure 4B), suggesting that the increase in eGFP expression levels was related to the increase in gene copy numbers (Figure 4C), which was consistent with previous studies (van Blokland et al., 2007).

### Analysis of Long-Term Stability of Transgene Expression

**FIGURE 5:**
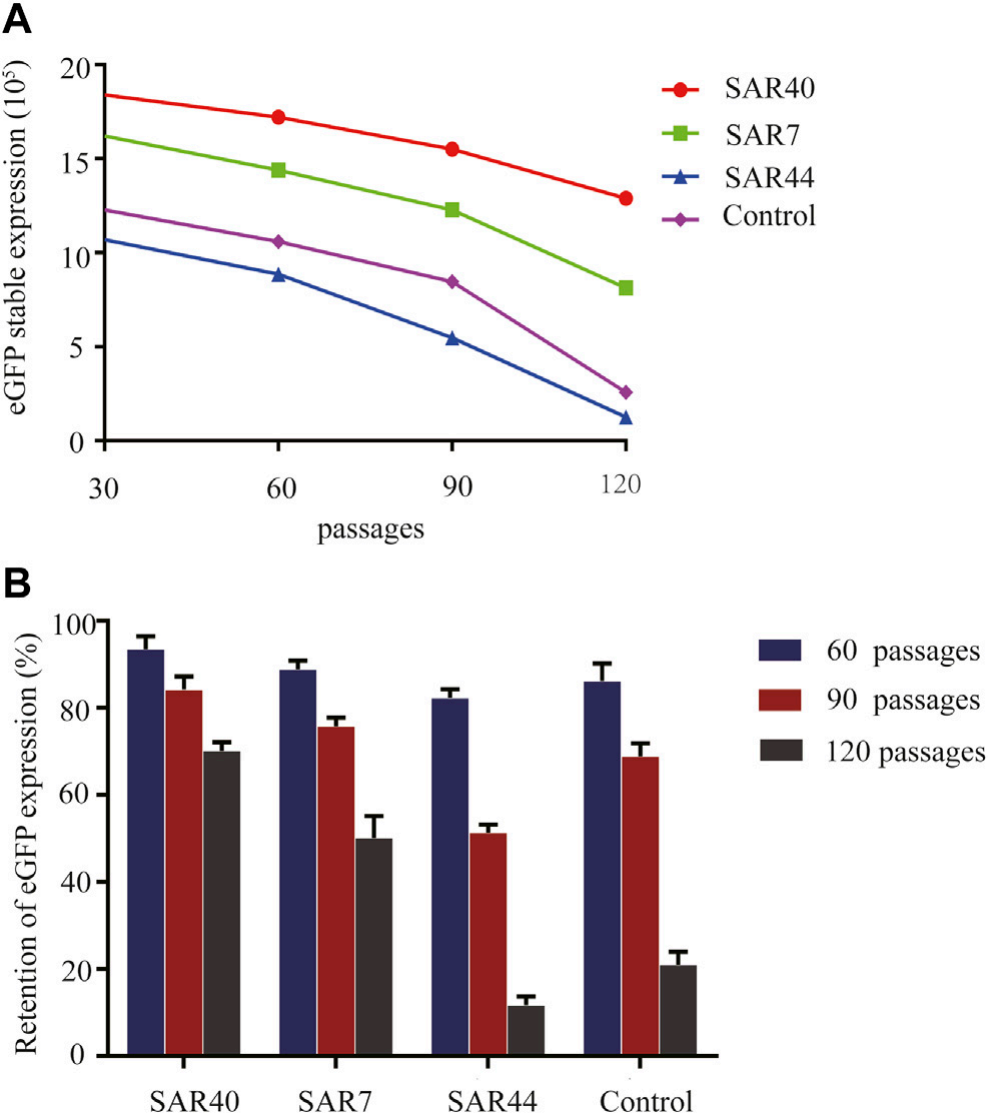
Analysis of long-term stability of the transgene expression in transfected CHO cells. Cells were collected, and FACSCalibur estimated the eGFP fluorescence profile at generation 30, 60,90, and 120 after transfection. **(A)** The eGFP expression (MFI) on different days after transfection. **(B)** The retention of eGFP expression of different constructs. The results are the mean values obtained for three independent experiments, and the standard deviation is indicated.

As it is very important to maintain the stability of transgene expression in CHO cells for a long time, we investigated the long-term stability at generation 120 after transfection. The cells were collected and the eGFP expression was analyzed at generations 30, 60, 90, and 120 post-transfection. As shown in Figure 5, SAR40 showed the highest eGFP expression, followed by SAR7 (Figure 5A), and the transgenic expression maintenance rate of SAR40 was the highest followed by that of SAR7 (Figure 5B), suggesting that SAR can help to maintain the stability of transgene expression.

### Analysis of Adalimumab Expression

**FIGURE 6:**
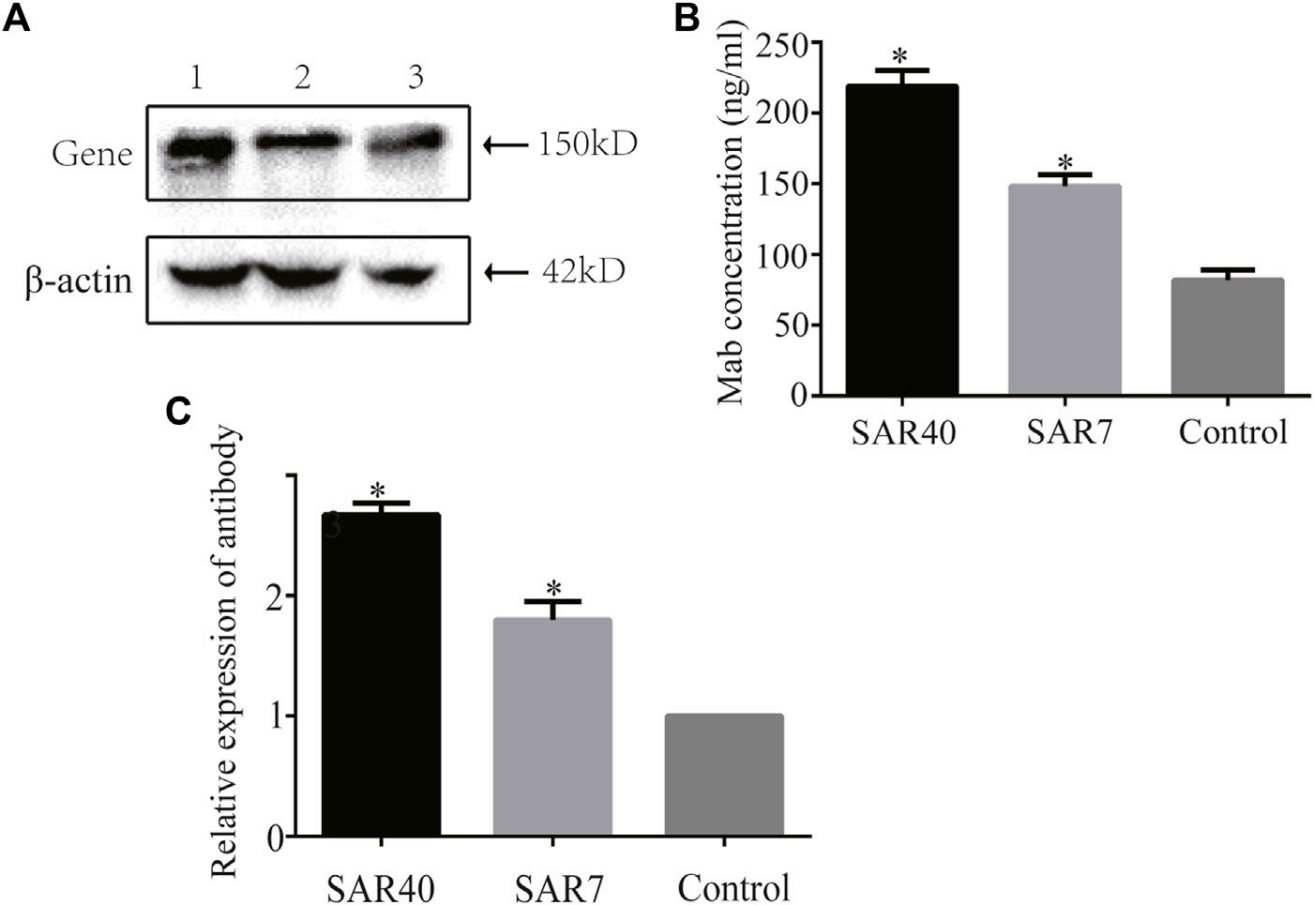
Analysis of adalimumab antibody expression of different constructs in CHO cells. The vectors containing adalimumab antibody gene were transformed into CHO cells, respectively. Cells supernatant was collected for analysis. **(A)** Adalimumab antibody expression was detected by Western blot. Lane 1, pIRES-mAb-SAR40; Lane 2, pIRES-mAb-SAR7; Lane 3, pIRES-mAb. **(B)** Expression of the adalimumab antibody was detected by ELISA. **(C)** Relative expression of adalimumab antibody. The results are the mean values obtained for three independent experiments, and the standard deviation is indicated (*p < 0.05).

We assessed the effect of SAR on secretory protein expression by analyzing the level of Adalimumab in stably transfected CHO cells cultured in serum-free suspension for 7 days. The cell supernatants were collected and the expression level of adalimumab was analyzed through Western blot. The results showed that adalimumab can be expressed in all vectors (Figure 6A). SAR40 and SAR7 can increase the Adalimumab expression levels 1.850 and 1.429 compared with the control in CHO cells (Figures 6B,C).

## Discussion

Recombinant therapeutic proteins have gained considerable attention in the biopharmaceutical industry. The large-scale industrial production of high-value therapeutic proteins is mainly achieved in mammalian cell lines, but this process is often hindered by low production and unstable expression (Kwaks and Otte, 2006; Jazayeri et al., 2018). Therefore, maintaining a high level of gene expression is a key factor affecting the production of recombinant proteins. The use of cis-epigenetic regulatory elements in the expression vector can result in more cell clones expressing proteins, as well as higher and more stable protein expression (Harraghy et al., 2015). In this study, we investigated the effect of SARs on transgenic expression and found that SARs can enhance the transgenic expression and maintain the long term expression of transgenes in transfected CHO cells, which may be used in the recombinant protein production in CHO cells.

SARs were discovered using a genomic screening strategy for elements that increase transgene expression, and are evolutionarily highly conserved at the sequence level, genome location, and function (Kwaks et al., 2003; Sautter and Enenkel, 2005). SAR is a blocker and not an enhancer of chromatin-related inhibitors. SAR can promote more cell clones expressing proteins, and confer a higher and more stable protein expression (Kwaks et al., 2003; van Blokland et al., 2007; Otte et al., 2007). Therefore, SAR has great significance in the application of recombinant protein production. In the present study, SARs were cloned upstream of the CMV promoter in the bicistronic expression vector, and the effects of three SARs on transgene expression were evaluated. eGFP is often used as a reporter gene to explore the regulatory effect of various cis-acting elements on gene expression (Seo et al., 2010; Wang et al., 2018); therefore, we first compared the transfection efficiency of different SARs using eGFP. We found that SAR7 and SAR40 showed the highest transfection efficiency, and their transient expression was stronger than that of SAR44 and the control vector, suggesting that the transfection efficiency is affected by SARs with different sequences. Moreover, the eGFP expression in the vectors containing SARs was indeed higher than that without SAR vectors, among which the eGFP expression for SAR7 and SAR40 was stronger than that for SAR44. Figure 2A is the representative photos observed by fluorescence microscopy and bright field image. The transfection efficiency of SAR44 is lower than that of the control, which is related to the vector structure. However, SAR44 has higher MFI than the control under the stably transfected state (Figure 3A), indicating that SAR44 can increase stably transgene in CHO cells. The increase in eGFP expression level correlated well with mRNA levels for the SAR-containing vectors and their gene copy numbers in a copy number-dependent manner, which was consistent with previous studies (Kwaks et al., 2003; Otte et al., 2007). However, SAR40-induced adalimumab expression was higher than that of SAR7. The effects of SAR on eGFP and mAb SAR are inconsistent, which may be due to eGFP is in one plasmid, but the mAb is coded by two plasmids. It is very important that the correct association of LC and HC during mAb production. The unbalanced proportion of LC to HC can cause low yields and aggregation. Another reason may be related to the secretory characteristics of the adalimumab antibody, and the exact mechanism needs further study. We hypothesize that the incorporation of SAR7 or SAR40 in the vectors may result in more colonies that produce higher gene expression levels. Moreover, SAR can interfere with the deacetylation or methylation of the surrounding chromatin, thus blocking the inhibitory effect of chromatin on the expression of exogenous genes at the molecular level (Kwaks et al., 2003).

During cell culture, the cell growth conditions can impact transgene expression. Cells change their gene expression mode according to different conditions such as nutrient consumption and shaking table speed (Sieck et al., 2014). Environmental stress has also been shown to cause epigenetic changes (Gim et al., 2015; Seo et al., 2015). Therefore, cell culture conditions may affect the epigenetic changes of cells, thus affecting the production of recombinant proteins.

In conclusion, in this study we explored the effects of SARs on transgene expression in CHO cells and found that SARs increase the transgenic expression in stably transfected CHO cells. SAR7 had the most remarkable effect, followed by SAR40. This effect may be caused by the increased gene copy numbers and their mRNA expression, but other factors need to be further explored. However, this study only compared three SARs in CHO cell line using eGFP and adalimumab. More SARs and transgenes should be used to investigate the effect of SAR elements on recombinant protein expression, for a successful industrial application.

## Data Availability

The datasets presented in this study can be found in online repositories. The names of the repository/repositories and accession number(s) can be found below: https://www.ncbi.nlm.nih.gov/genbank/, AY190751.1, AY190756.1.
